# Disease Management and the Internet

**DOI:** 10.2196/jmir.6.3.e33

**Published:** 2004-09-29

**Authors:** George Demiris

**Affiliations:** ^1^Department of Health Management and InformaticsUniversity of Missouri-ColumbiaColumbia MOUSA

This issue contains three articles on the use of the Internet in disease management, the anticipated benefits and challenges associated with this concept, and emerging trends. The studies presented in this issue aim to determine whether/how the use of Web-based applications can assist in managing chronic conditions over time, improve clinical outcomes and lower costs both through patient education and physiological monitoring.

The health care sector is facing challenges such as rapidly-escalating medical costs, growing life expectancy rates and expanding segments of the population suffering from chronic conditions such as diabetes, obstructive pulmonary disease, and congestive heart failure. Several disease management initiatives aim to reduce the rate of inpatient hospitalization, the use of emergency room services, and the number of physician office visits—innovations that would allow people to stay at home enjoying increased quality of life and independence. A possible reduction of utilization rates would also lower both clinical and administrative costs.

Several challenges are associated with the diffusion of Web-based disease management initiatives. Such initiatives require an infrastructure that will enable efficient and timely communication among health care providers, case managers, health plan staff, and caregivers, and that will enable coordination of all related services. One of the most important factors, which will greatly affect the growth of such systems, is, obviously, reimbursement. In the United States in the year 2000, First Health, a national health benefits company based in Illinois, was one of the first health plans in the country to reimburse providers for electronic communication with patients under specific circumstances. Since then, additional insurance companies and health plans have been redefining their reimbursement policies for online services. In 2003 Blue Shield of California started reimbursing physicians for online consultations.

Many believe that the Internet can enhance a shift from institution-centric to patient-centric systems that empower individuals with chronic conditions to play an active role in the management of their diseases. A large portion of the population diagnosed with chronic conditions, however, is of lower socioeconomic status. Concerns have been expressed that these citizens might have limited access to the Internet and other electronic applications, and as a result might be excluded from disease management systems due to the so-called digital divide.

In this issue, three papers introduce new concepts for Web-based disease management or specific applications that have been pilot tested. One of these applications described by Anhøj and Nielsen is LinkMedica, a Web service for asthma patients and health care professionals [1]. Link Medica enables asthma patients to monitor their conditions using an online diary and enables health care providers to access the patients' diary data. The study describes the first three years of this project and outlines the reasons that patients seemed to underutilize the Web service after short periods. The study reports that patients felt after a while that the system was not easily integrated into their daily schedule. Issues of time and inconvenience as well as psychological factors were addressed. These findings raise an important ethical concern that is associated with the daily use of Web-based in-home applications for patients with chronic conditions, namely, the “medicalization” of the home environment or what Bauer calls turning the home into a “*de facto* ICU” [2]. Daily use of systems over longer periods of time are sometimes perceived as psychologically burdensome to patients. The second study by Anhøj and Jensen focuses on the use of the Internet as a tool to enhance lifestyle changes concerning diet and physical activity [3]. Patients and practitioners seem to appreciate such applications but the study illustrates the challenges for their successful implementation. One of the great challenges is the usability of the system interfaces. Furthermore, the study indicates that selecting the type and amount of information that become included in patient education systems needs to be customized to the specific needs of the target audience. Finally, the third paper by Wiecha and Pollard introduces us to the concept of an interdisciplinary e-health team [4]. The Internet is an appropriate platform for supporting interdisciplinary clinical teamwork. The authors argue that teamwork supported by properly-designed e-health applications could help create more effective systems of care for chronic disease.

As we discussed in the call for papers for this issue, the factors that will be critical for the diffusion of Internet-based disease management systems include the usability of the design, issues of privacy and confidentiality of data, patient and provider acceptance, development and maintenance costs, and reimbursement structures [5]. The studies presented in this issue provide us with a better understanding of these challenges and ways to address them.
